# Thermo-Sensitive TRP Channels: Novel Targets for Treating Chemotherapy-Induced Peripheral Pain

**DOI:** 10.3389/fphys.2017.01040

**Published:** 2017-12-13

**Authors:** Mustafa Nazıroğlu, Nady Braidy

**Affiliations:** ^1^Neuroscience Research Center, Suleyman Demirel University, Isparta, Turkey; ^2^Centre for Healthy Brain Ageing, School of Psychiatry, University of New South Wales, Sydney, NSW, Australia

**Keywords:** allodynia, chemotherapeutic agents, hyperalgesia, oxidative stress, thermo sensitive TRP channels

## Abstract

Abnormal Ca^2+^ channel physiology, expression levels, and hypersensitivity to heat have been implicated in several pain states following treatment with chemotherapeutic agents. As members of the Ca^2+^ permeable transient receptor potential (TRP), five of the channels (TRPV1-4 and TRPM2) are activated by different heat temperatures, and two of the channels (TRPA1 and TRPM8) are activated by cold temperature. Accumulating evidences indicates that antagonists of TRPA1 and TRPM8 may protect against cisplatin, oxaliplatin, and paclitaxel-induced mitochondrial oxidative stress, inflammation, cold allodynia, and hyperalgesia. TRPV1 was responsible from the cisplatin-induced heat hyperalgesia and mechanical allodynia in the sensory neurons. TRPA1, TRPM8, and TRPV2 protein expression levels were mostly increased in the dorsal root (DRG) and trigeminal ganglia by these treatments. There is a debate on direct or oxaliplatin-induced oxidative cold stress dependent TRPA1 and TRPV4 activation in the DRG. Involvement of molecular pathways such as cysteine groups, glutathione metabolism, anandamide, cAMP, lipopolysaccharide, proteinase-activated receptor 2, and mitogen-activated protein kinase were also indicated in the oxaliplatin and paclitaxel-induced cold allodynia. In this review, we summarized results of five temperature-regulated TRP channels (TRPA1, TRPM8, TRPV1, TRPV2, and TRPV4) as novel targets for treating chemotherapy-induced peripheral pain

## Introduction

Chemotherapeutic agents such as taxanes (paclitaxel, docetaxel) and platinum analogs (cisplatin, carboplatin, oxaliplatin) are used in treatment of several cancer types. Taxanes inhibit progression of mitosis through stabilization of tubulin in the treatment of solid tumors (Sharma et al., 2007). However, platinum derived chemotherapeutic drugs inhibit DNA synthesis and repair through cross-linking of DNA strands and are used for the treatment of several cancer types such lung carcinoma, testicular cancer, ovarian cancer, etc. (Kelland, 2007). However, severe painful neuropathy is a main complication of these cancer agents. Several peripheral neuropathies such as numbness, tingling, and chronic pain distributed in a distal stocking-and-glove pattern have been reported in patients treated with a variety of chemotherapeutic agents. The etiology of painful neuropathy remains unclear. Current analgesic drugs cannot completely alleviate the pain, although they may provide partial analgesic effects in some patients. Severe painful neuropathy due to chemotherapeutic agents has pushed some patients to suicide (Lester and Yang, 1996; Bauduer et al., 2000). Therefore, discovery of novel therapeutic agents against chemotherapy-induced painful neuropathy is an urgent subject.

In the etiology of pain and neuropathy, calcium ion (Ca^2+^) overload plays an important role. Ca^2+^ enters into cells by different ways including cation channels. Voltage gated calcium channels (VGCC) and chemical channels (i.e., glutamate) are well-known calcium channels (Kumar et al., 2014). However, new calcium channels namely the transient receptor potential (TRP) superfamily were discovered in eye cells of drosophila flys (Hardie, 2011; Nazıroğlu, 2011). Today, the TRP superfamily contains 28 channels with 7 different subgroups (Nazıroğlu, 2011; Uchida et al., 2017).

Dorsal root ganglion (DRG) neurons have important roles in the pathobiology of neuropathic pain. There is no barrier between the DRG and blood, and compounds with a high molecular weight can easily diffuse into the DRG (Abram et al., 2006). The TRPA1, TRPV1 and TRPV4 channels are mainly expressed in the DRG and trigeminal ganglia neurons (Kobayashi et al., 2005; Obata et al., 2005; Fonfria et al., 2006; Nativi et al., 2013; Yazğan and Nazıroğlu, 2017). Hence, the TRPA1, TRPV1 and TRPV4 have been associated with pain transmission of sensory neurons, including the DRG (Materazzi et al., 2012; Kahya et al., 2017).

Some peripheral primary afferent fibers are affected by low and high temperature changes and are called thermoreceptors. So far, 11 TRP channels in mammalian cells have been identified as thermosensitive TRP (thermo-TRP) channels (Uchida et al., 2017). Two TRP channels (TRPV1 and TRPV2) are activated by high temperatures (43°C≥ and 55°C≥, respectively). Five TRP channels (TRPV1-4 and TRPM2) are activated by different heat temperatures, although two of TRP channels (TRPA1 and TRPM8) are activated by cold (≤17°C) and (≤25°C) cool temperatures, respectively (Caterina et al., 1999; Xu et al., 2002; Story et al., 2003; Bandell et al., 2004; Nazıroğlu and Ozgül, 2012). In addition, the remaining **two** channels, TRPM3 and TRPC5 are noxious heat and cold sensors, respectively (Vriens et al., 2011; Zimmermann et al., 2011). In addition, TRPV1, TRPA1, and TRPV4 are also oxidative stress-sensitive Ca^2+^-permeable channels. Therefore, activation of TRPA1 and TRPV4 in neurons by oxidative stress such as H_2_O_2_ has been previously reported (Bai and Lipski, 2010; Materazzi et al., 2012; Toda et al., 2016). However, activation of TRPA1 and TRPV4 in the DRG neurons was inhibited by members of the cysteine antioxidant redox cycle such as glutathione (GSH) and selenium (Materazzi et al., 2012; Kahya et al., 2017).

Owing to the high expression levels of TRPA1, TRPM8, TRPV1, TRPV2, and TRPV4 in the DRG (Bridges et al., 2003; Obata et al., 2005; Fonfria et al., 2006; Nativi et al., 2013), these channels represent novel targets for the management of chemotherapy-induced neuropathic pain. Present information on chemotherapy-induced neuropathic pain in human and experimental animals suggests the involvement of at least five thermo-TRP channels (TRPA1, TRPM8, TRPV1, TRPV2, and TRPV4; Table 1). In this review, we summarized the potential role of the five thermo TRP channels as novel targets for treating chemotherapy-induced neuropathic pain.

**Table 1:** Roles of TRPA1, TRPM8, TRPV1, TRPV2, and TRPV4 channels on chemotherapeuty-induced peripheral pain in experimental animals.

**Channel**	**Agent**	**Material**	**Value/Effect**	**References**
TRPA1	Oxaliplatin	Rat DRG	Increased cAMP level and channel sensitization	Anand et al., 2010
TRPA1	Oxaliplatin	Mice DRG- trigeminal ganglion	Increased channel expression level	Ta et al., 2010
TRPA1	Oxaliplatin	Mice DRG and CHO	Oxidative and cold allodynia	Nassini et al., 2011
TRPA1	Oxaliplatin	Mice DRG	Increased channel sensitization but no effect on cold hyperalgesia	Chen et al., 2011
TRPA1	Oxaliplatin	Mice DRG	Increase of channel protein expression level but no change of cold hypersensitivity	Descoeur et al., 2011
TRPA1	Oxaliplatin	Mice DRG and CHO	Oxidative and cold allodynia	Materazzi et al., 2012
TRPA1	Oxaliplatin	Mice DRG	No thermal hyperalgesia but cold allodynia	Park et al., 2015
TRPA1	Oxaliplatin	Rat DRG	increase of PAR2 and channel activation	Tian et al., 2015
TRPA1	Oxaliplatin	Rat DRG	Increase of nocifensive behaviors and channel mRNA expression levels	Mizuno et al., 2014
TRPA1	Oxaliplatin	Mice DRG	Increase of channel protein expression and cold hypersensitivity	Yamamoto et al., 2016
TRPA1	Oxaliplatin	Mice DRG	Decrease of oxaliplatin-induced TRPA1 expression, cell death and neuropathic pain by GSH treatment	Lee et al., 2017
TRPA1	Cisplatin	Mice DRG- trigeminal ganglion	Increased channel expression level	Ta et al., 2010
TRPA1	Paclitaxel	Rat DRG	TRPA1-stimulated transmitter release was increased or decreased as concentration and exposure time dependent	Pittman et al., 2014
TRPM8	Cisplatin	Mice DRG- trigeminal ganglion	Increased channel expression level	Ta et al., 2010
TRPM8	Oxaliplatin	Mice DRG	Increase of channel expression and cold allodynia	Gauchan et al., 2009
TRPM8	Oxaliplatin	Mice DRG- trigeminal ganglion	No change channel expression level	Ta et al., 2010
TRPM8	Oxaliplatin	Mice DRG	No change in the TRPM8 protein expression but increase of cold hypersensitivity	Descoeur et al. (2011)
TRPM8	Oxaliplatin and oxalate	Rat DRG	Increase of TRPM8 mRNA and protein expression levels	Kawashiri et al., 2012
TRPM8	Oxaliplatin	Rat DRG	Increase of nocifensive behaviors and channel mRNA expression levels	Mizuno et al., 2014
TRPV1	Cisplatin	Mice	Mechanical hyperalgesia but no pronociceptive role of TRPV1 in toxic neuropathy	Bölcskei et al., 2005
TRPV1	Cisplatin	Mice DRG- trigeminal ganglion	Increased TRPV1 protein expression level	Ta et al., 2010
TRPV1	Cisplatin	Rat DRG	No changes in TRPV1 protein expression	Hori et al., 2010
TRPV1	Cisplatin	Mice DRG	Increase of TRPV1 protein expression	Khasabova et al., 2012
TRPV1	Oxaliplatin	Mice DRG- trigeminal ganglion	No change channel expression level but thermal hyperalgesia and mechanical allodynia	Ta et al., 2010
TRPV1	Oxaliplatin	Mice DRG	Increase of channel sensitization but no effect on cold hyperalgesia	Chen et al., 2011
TRPV1	Oxaliplatin	Mice DRG	Increased channel sensitization	Wainger et al., 2015
TRPV1	Paclitaxel and vinorelbine	Rat DRG	No effect on paclitaxel and vinorelbine-induced substance P production	Miyano et al., 2009
TRPV1	Paclitaxel	Mice DRG	Increase of channel expression and thermal hyperalgesia.	Hara et al., 2013
TRPV1	Paclitaxel	Rat DRG	TRPV1-stimulated transmitter release was increased or decreased as concentration and exposure time dependent	Pittman et al., 2014
TRPV1	Paclitaxel	Human and rat DRG	TRPV1 stimulation via Toll-like receptor 4 signaling	Li et al., 2015
TRPV1	5-FU	Rat	Channel activation and pain induction by TRPV1 but not TRPA1	Chen et al., 2013
TRPV2	Cisplatin	Rat DRG	Increased TRPV2 protein expression in the small DRG but not in DRG innervating gastrocnemius muscle	Hori et al., 2010
TRPV4	Paclitaxel	Rat DRG	Hyperalgesia though activation of α2β1 integrin and Src tyrosine kinase pathways	Alessandri-Haber et al., 2008
TRPV4	Paclitaxel	Rat DRG	No effect	Alessandri-Haber et al., 2004
TRPV4	Oxaliplatin	Mice DRG	Increase of channel sensitization but no effect on cold hyperalgesia	Chen et al., 2011
TRPV4	Oxaliplatin	Mice DRG	Oxidative but not cold allodynia	Materazzi et al., 2012

## Chemotherapeutic agents

**Figure 1:**
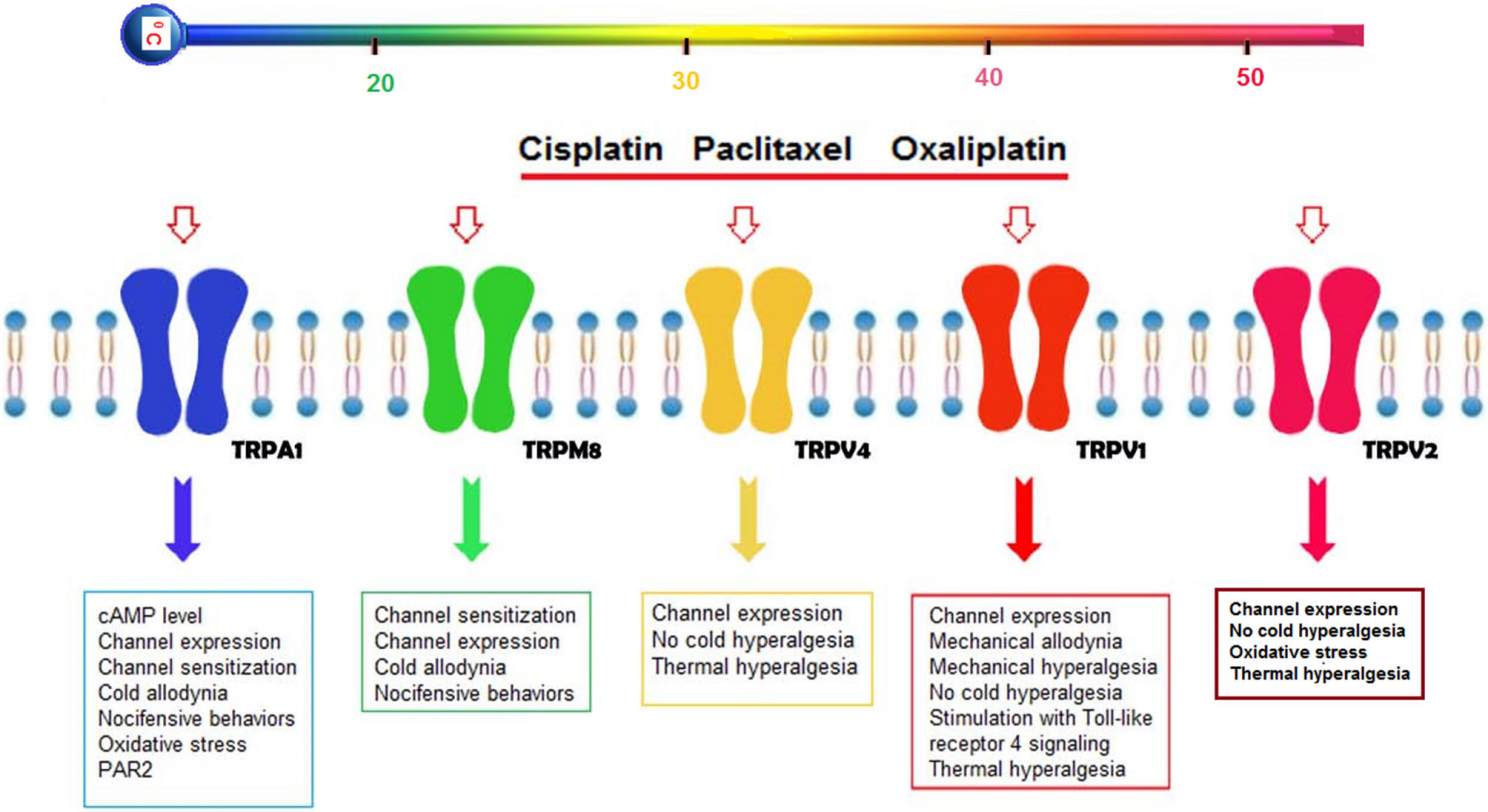
Possible effects of cisplatin, oxaliplatin, and paclitaxel on thermo-TRP channels (TRPA1, TRPM8, TRPV1, TRPV2, and TRPV4) in the DRG neurons. Three chemotherapeutic agents (cisplatin, oxaliplatin, and paclitaxel) induce severe peripheral pain adverse effect in treatment of cancer patients. Reports on chemotherapy-induced pain in peripheral nerves were focused on five thermo-TRP channels (TRPA1, TRPM8, TRPV1, TRPV2, and TRPV4), because their expression levels are mostly high in the peripheral neurons. Activation of the five thermo-TRP channels by the cisplatin, oxaliplatin and paclitaxel lead to changes on levels of channel expression, channel sensitization, nociceptive behaviors, oxidative stress, mechanical, heat and cold hypersensitivity (Anand et al., 2010; Ta et al., 2010; Hara et al., 2013). In addition, the levels are induced by activation of some secondary molecular mechanisms such as glutathione (GSH) (Lee et al., 2017), proteinase-activated receptor 2 (PAR2) (Tian et al., 2015), cAMP (Anand et al., 2010), and Toll-like receptor 4 (TLR4) signaling (Meseguer et al., 2014).

There is limited information on chemotherapeutic agents and thermo-TRP channels in pain induction, although the mode of action of chemotherapeutic agents has been well-established (Figure 1).

### Cisplatin

Cisplatin is an effective anti-tumor drug that is used in the treatment of several cancers such as ovarian, bladder, and testicular cancers (Kelland, 2007). It acts by crosslinking DNA it inhibits DNA replication and repair mechanisms through formation of DNA-platinum products (Dzagnidze et al., 2007). However, it also induces several adverse effects such as mechanical allodynia, hyperalgesia, and toxicity in neurons including DRG. Cisplatin produces a cumulative toxic effect on peripheral nerves, and 30–40% of cancer patients receiving this agent experience neuropathic pain (Khasabova et al., 2012). Apoptosis, oxidative stress and necrosis pathways in the cancer cells through TRP channel stimulation are also activated by cisplatin treatment (Sakalli Çetin et al., 2017). Cisplatin-induced neuropathy and apoptosis of sensory neurons were attributed to uptake of the drug into the DRG affecting large myelinated sensory nerve fibers (Ta et al., 2006). The damage and injury to DRG neurons could be partially decreased by different adjuvant therapies such as anandamide (as an endogenous cannabinoid), which attenuates hyperalgesia in neuropathic pain (Khasabova et al., 2012). Therefore, the prevention or reduction in the neurotoxic effects of cisplatin remains of major clinical significance in cancer patients.

### Oxaliplatin

Oxaliplatin is a platinum complex containing agent and is the most commonly using anti-tumor agent for the treatment of several cancer types such as colorectal cancer (Kelland, 2007). Oxaliplatin has less complications than cisplatin, including a lower incidence of hematotoxicity and manageable gastrointestinal toxicity compared to other platinum-based chemotherapeutics. However, neurotoxicity remains a very common complication in patients treated with oxaliplatin, because it has a long terminal half-life (Descoeur et al., 2011; Zhao et al., 2012). Acute and chronic pain has been reported in patients treated with oxaliplatin. Acute neuropathies are characterized as accrual numbness, paresthesia, dysesthesia, and peripheral pain following 1–12 h of treatment. One of main complication in the treatment of oxaliplatin in the cancer patients is increased cold sensitivity (Kelland, 2007). Oxaliplatin is metabolized to oxalate, and dichloro (1,2-diaminocyclohexane)platinum are produced during the metabolism of oxaliplatin (Nakagawa and Kaneko, 2017). Cold hyperalgesia and mechanical allodynia of oxaliplatin is attributed to oxalate and dichloro (1,2-diaminocyclohexane)platinum (Sakurai et al., 2009; Nakagawa and Kaneko, 2017). Additionally, these metabolites are also responsible for oxaliplatin-induced cold oxidative stress (Nakagawa and Kaneko, 2017).

### Paclitaxel

One of the most common chemotherapeutic agents is paclitaxel which was originally isolated from Pacific Yew tree *Taxus brevifolia* Nutt (Wani et al., 1971). Paclitaxel has been mostly used in treatment of lung, ovarian, head, neck and breast cancer (Chen et al., 2011). In paclitaxel treatment, the division of cancer cells is inhibited through dynamic assembly or disassembly of the mitotic spindle (Marupudi et al., 2007). Hypersensitive reactions such as bronchospasm, pulmonary edema and neuropathy occur during treatment with paclitaxel (Shepherd, 2003; Sisignano et al., 2016). Recent studies have suggested the involvement of mitochondrial oxidative stress and overload Ca^2+^ entry through VGCC and TRP channels (Materazzi et al., 2012; Duggett et al., 2016; Sekiguchi et al., 2016), although the exact mechanism of neuropathic pain induced by paclitaxel remains to be elucidated.

## Chemotherapeutic agents and thermo-TRP channels

**Figure 2:**
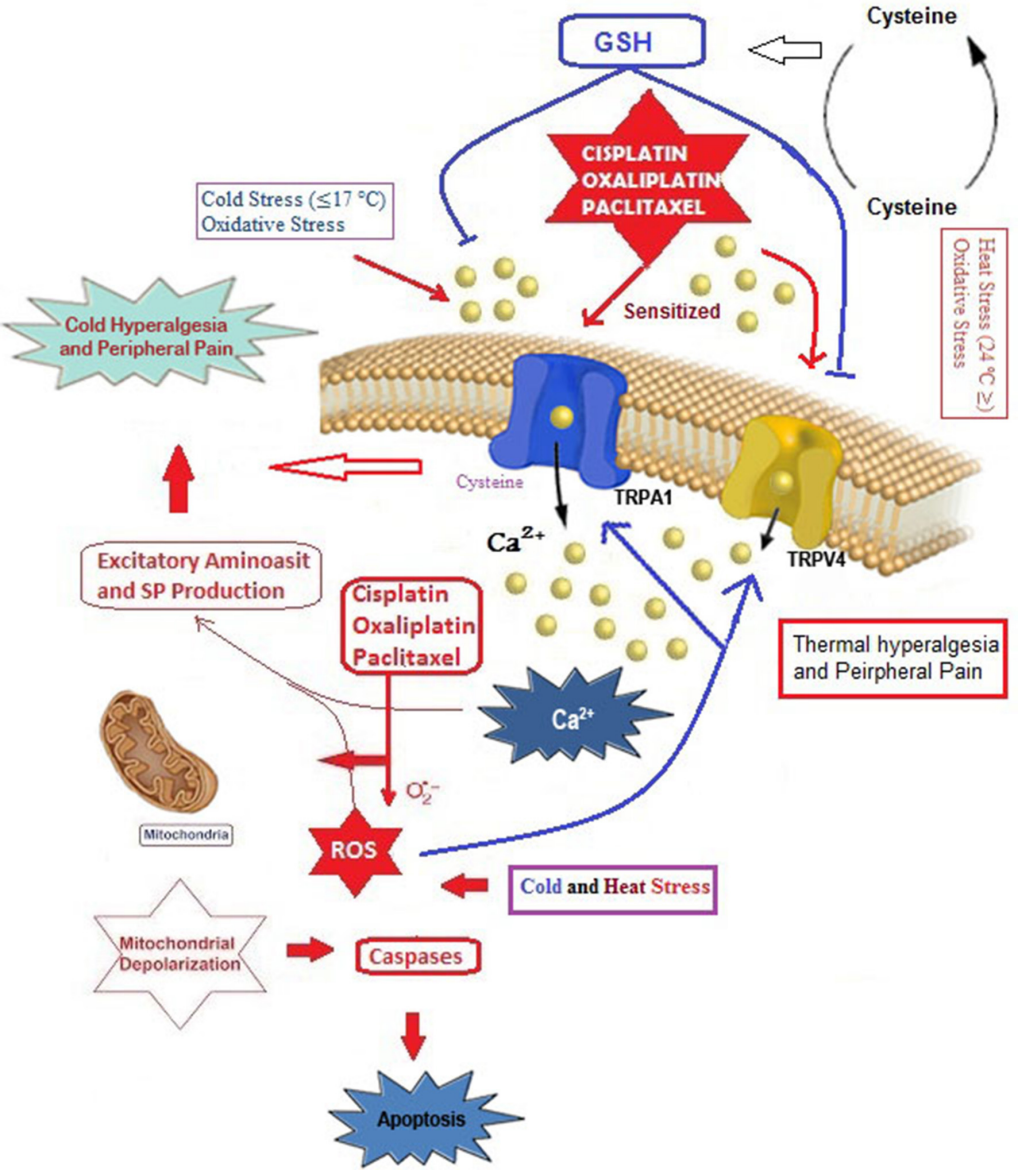
Possible molecular pathways of cisplatin, oxaliplatin and paclitaxel on oxidative stress-dependent TRPA1 and TRPV4 activation in the DRG neurons. Cysteine groups are main target of oxidative stress in cellular membranes and membrane of TRPA1 has rich content of cysteine groups (Takahashi et al., 2011). TRPA1 and TRPV4 are oxidative stress-sensitive Ca^2+^-permeable channels. The cisplatin, oxaliplatin, and paclitaxel can results in augmented TRPA1 and TRPV4, leading to Ca^2+^ influx through direct channel activation or excessive production of oxidative stress and induction of apoptosis through depolarization of mitochondrial membranes. Overload Ca^2+^ influxes induce pain through substance P (SP) and excitatory amino acid production. Glutathione (GSH) is synthetized from cysteine redox cycle. Protective role of GSH on TRPA1 and TRPV4 through oxaliplatin and paclitaxel-induced oxidative stress in DRG neuron was reported (Materazzi et al., 2012). The molecular pathway may be a cause of chemotherapy-induced peripheral pain and this subject warrants further investigation.

As already mentioned, chemotherapeutic agent can cause painful neuropathy that is usually resistant to analgesic drugs (Hara et al., 2013; Oehler et al., 2017). In addition to chronic neuropathy, paclitaxel is also associated with an acute pain syndrome (Chen et al., 2011), although its exact mechanism remains unclear. Accumulating evidence on chemotherapy-induced pain and hypersensitivity through activation of cation channels such as TRPA1, TRPM8, TRPV1, and TRPV4 focused on two main subjects, oxidative stress, and Ca^2+^ overload (Figure 2).

### TRPA1

TRPA1 is a member of ankyrin subfamily in the TRP superfamily. There are 6 domains and 4 pores in the structure of the TRPV1 channel. TRPV1 is activated by different stimuli such as oxidative stress, chemicals such as mustard oil and cinnamaldehyde, and cold body temperature (≤17°C).

Excessive reactive oxygen species (ROS) and low levels of antioxidants play a pivotal role in the pathobiology of cancers (Koçer and Nazıroğlu, 2013; Koçer et al., 2014). As already mentioned, the TRPA1 and TRPV4 channels are activated by different stimuli, including oxidative stress (Bai and Lipski, 2010). Involvement of cysteine residues and the antioxidant, dithiothreitol in the N domain of TRPA1, were indicated by a mass spectrometry study (Macpherson et al., 2007). Activation of TRPA1 though reversible covalent or oxidative modifications of the cysteine residues in DRG of wild and TRPA1 knockout mice were reported (Andersson et al., 2008; Salazar et al., 2008). Activations of TRPA1 and TRPV4 were reported in the DRG of wild type and TRPA1 knockout mice by cold exposure and paclitaxel-induced excessive reactive oxygen species (ROS) production and mechanical allodynia, although the allodynia and oxidative stress was partially decreased by the TRPA1 (HC-030031) and TRPV4 (HC-067047) antagonist treatments (Materazzi et al., 2012). However, another study did not observe significant differences in calcium response as an effect of oxaliplatin or cisplatin exposure in cultured mouse DRG and naïve Chinese hamster ovary (CHO) cell line (Nassini et al., 2011), although cisplatin and oxaliplatin evoked an antioxidant GSH-sensitive calcium response in the CHO cell line and DRG neuron. Result of another study indicated that a single dose of oxaliplatin produced mechanical and cold hyperalgesia in wild type rats. In addition, mechanical and cold allodynia were induced in wild type mice but not in TRPA1 knockout mice (Nassini et al., 2011). The study also reported that cisplatin and oxaliplatin caused TRPA1 activation via excessive ROS production instead of direct channel targeting (Nassini et al., 2011). In addition, it was reported that cisplatin and oxaliplatin-induced neuropathy was treated through inhibition of platinum accumulation in the DRG of rats (Holmes et al., 1998) and patients with cancer, using GSH (Cascinu et al., 2002). Result of a recent study indicated that oxaliplatin-induced increase in TRPA1 expression, cell death and neuropathic pain in the DRG of mice were decreased by treatment with aluminum and GSH (Lee et al., 2017).

In addition to cysteine oxidation in the N domain of TRPA1, inhibition of prolyl hydroxylases pathway through decreased oxygen levels on the activation of TRPA1 in the vagal and sensory neurons of mice was also reported (Takahashi et al., 2011). The inhibition of prolyl hydroxylase (PHD) induced hydroxylation of a proline residue in the N-terminal ankyrin repeat domain induces activation of TRPA1 through induction of hypoxia (Nakagawa and Kaneko, 2017). TRPA1 activation through PHD inhibition on oxaliplatin-induced cold hypersensitivity has been previously investigated. The study showed oxaliplatin, and dimethyl oxalate as a membrane-permeable oxalate analog induced TRPA1 sensitization to ROS by inhibiting PHD -mediated hydroxylation of the Pro394 residue on human TRPA1 (Miyake et al., 2016).

During inflammation, p38 mitogen-activated protein kinase (MAPK) has a significant role in the development and maintenance of neuropathic pain. The involvement of TRPA1 through activation of p38 MAPK in oxaliplatin-induced acute cold hypersensitivity in mice DRG neuron was recently reported (Yamamoto et al., 2016). The involvement of N-formylmethionine peptides such as formylmethionyl-leucyl phenylalanine [fMLP] in the induction of acute pain and mechanical allodynia through activations of TRPA1 and TRPV1 in mice were indicated by fMLP treatment (Chiu et al., 2013). Lipopolysaccharide (LPS) is a toxic by-product of bacterial lysis and mechanical allodynia is induced through activation of TRPA1 and through activation of the Toll-like receptor 4 (TLR4) signaling pathways in mice exposed to LPS treatment (Meseguer et al., 2014).

Hypersensitivity to mechanical stimuli is called “mechanical allodynia,” while a thermal stimulus is called “thermal hyperalgesia.” Chemotherapy-induced peripheral neuropathy has been widely investigated in experimental animals as mechanical allodynia and thermal hyperalgesia. Results of TRPA1, TRPM8, and TRPV1 on mechanical allodynia and thermal hyperalgesia are conflicting. For example, induction of a cold hypersensitivity through activation of TRPM8 but not TRPA1 in mice DRG neurons was reported following acute oxaliplatin treatment (Descoeur et al., 2011). However, on the contrary, the involvement of TRPA1 but not TRPM8 and TRPV1 was reported by Zhao et al. (2012) in oxaliplatin induced acute neuropathy in DRG neurons. No significant difference was also reported between oxaliplatin and vehicle groups for thermal hyperalgesia at 42, 47, and 52°C, although the presence of cold allodynia through TRPA1 activation was reported in oxaliplatin-treated mice (Park et al., 2015).

Proteinase-activated receptor 2 (PAR2) is a member of PAR subfamily of G protein-coupled receptors and activation of these receptors regulates several pathophysiological processes including inflammation and pain (Wu et al., 2017). The role of PAR2 on oxaliplatin-induced TRPA1 activation and peripheral pain induction was recently investigated in rat DRGs by Tian et al. (2015). The induction of mechanical hyperalgesia and cold hypersensitivity through increased PAR2 activation was also reported in the same study (Tian et al., 2015). Similarly, it has been demonstrated that inhibition of PAR2 increased oxaliplatin-induced cold sensitivity, and blockade of the TRPV1 channel induced little effects on oxaliplatin-induced cold hypersensitive in superficial dorsal horn of the rat spinal cord (Chen et al., 2015). The results of both studies suggest the involvement of PAR2 in TRPA1 activation induced cold allodynia, but not TRPV1-induced cold hypersensitive in oxaliplatin-treated rats. In addition, increase of channel sensitizations in TRPA1, TRPV1, and TRPV4 was reported in DRG of mice by paclitaxel treatment, although paclitaxel-induced cold hyperalgesia was not decreased by treatment with TRPA1, TRPV1, and TRPV4 channel antagonists (Chen et al., 2011).

The involvement of increased intracellular cAMP levels on TRP sensitization mediated neuronal damage was reported (Anand et al., 2010). Results of several studies indicated that cancer patients are very sensitive to cold after oxaliplatin treatment. In addition to chemicals and oxidative stress, cold body temperature (≤17°C) activates TRPA1. Therefore, the TRPA1 acts as a “cold sensor” which is increased by pain induction (Yamamoto et al., 2016), although there is inconsistent evidence for its role in cold detection (Bandell et al., 2004; Bautista et al., 2005; Anand et al., 2010). In addition, there is synergic interaction between TRPA1 and TRPV1 on channel activation mechanisms in DRG, because TRPA1 is colocalized with 30–50% TRPV1 expressing neurons in rat and human DRG (Bautista et al., 2005). The sensitization ratio of TRPA1 and TRPV1 are affected by several factors, including chemotherapeutic agents. Increased TRPA1 and TRPV1 channel sensitization was reported in peripheral neurons of oxaliplatin-treated mice (Anand et al., 2010; Wainger et al., 2015). Increased protein expression levels of TRPA1 (Ta et al., 2010; Descoeur et al., 2011; Nassini et al., 2011; Zhao et al., 2012; Yamamoto et al., 2016), TRPV1 (Descoeur et al., 2011; Nassini et al., 2011), and TRPM8 protein (Gauchan et al., 2009; Ta et al., 2010; Descoeur et al., 2011) were reported in DRG and trigeminal ganglion by acute oxaliplatin and cisplatin treatments, but conflicting reports are also present on the expression levels of TRPA1, TRPM8 (Zhao et al., 2012), and TRPV1 (Ta et al., 2010; Zhao et al., 2012) in the DRG neurons.

Goshajinkigan is a traditional Japanese medicine and it has been previously used for the treatment of numbness of the extremities, low back pain, and diabetic neuropathy. The effect of Goshajinkigan on neuropathy through inhibition or stimulation of TRPA1, TRPM8, and TRPV1 channels in oxaliplatin-induced neuropathy rat model was recently investigated (Mizuno et al., 2014). Enhanced nociceptive behaviors and DRG mRNA expression levels of TRPA1 and TRPM8 but not TRPV1 mRNA in the oxaliplatin-treated rats were also decreased following treatment with goshajinkigan.

There are some notable differences on cold dependent-activation of TRPA1 channel between humans and rodents. For example, cold dependent activation of TRPA1 was reported in rat and mouse but not in humans or rhesus monkeys (Chen et al., 2013). Co-expression and synergic interactions between TRPV1 and TRPA1 were also observed in nociceptive neuronal fibers in rats with oral ulcerative mucositis–induced spontaneous pain following treatment with 5-fluorouracil (5-FU), and the TRPV1 but not TRPA1 was activated by 5-FU treatment (Chen et al., 2013).

### TRPM8

TRPM8 is expressed in a distinct subset of nociceptors, including DRG neurons and the channel is activated by cool temperature (<25°C), menthol, icilin (Nazıroğlu and Ozgül, 2012; Okazawa et al., 2014). As already mentioned, hypersensitivity of cold stimuli in patients can occur after infusion of oxaliplatin into cancer patients. Oxaliplatin induced-cold allodynia and increases in TRPM8 mRNA levels in the DRG of rats were also reported (Gauchan et al., 2009; Ta et al., 2010). However, oxaliplatin-induced cold hypersensitivity in neuropathic pain models were decreased by deletion of the TRPM8 gene and treatments of TRPM8 and TRPV1 antagonists, but not by a TRPV1 antagonist (5′-iodoresiniferatoxin) treatment (Gauchan et al., 2009; Ta et al., 2010). Consistent with these reports, one study reported oxaliplatin-induced induction of cold hyperalgesia and increased TRPM8 mRNA levels (3, 5, and 8 days of oxaliplatin treatment) in the DRG of rats (Kawashiri et al., 2012). Furthermore, they observed oxalate-induced increase of TRPM8 protein in the DRG (Kawashiri et al., 2012).

Voltage gated calcium channels (VGCC) are very selective to Ca^2+^ and they are activated by increases in voltage but they are inhibited by a decrease of intracellular and cell membrane voltage changes. Based on their threshold of voltage-dependent activation, they were divided into two subgroups as high-voltage activated channels (HVA) and low-voltage-activated (LVA) channels (Kumar et al., 2014). HVA channels can be further subdivided into 5 types (L-, P/Q-, N-, and R-type) according to their biophysical, pharmacological, and molecular features. Interactions between TRPM8 and molecular pathways or other calcium channels were also investigated in DRG neurons of oxaliplatin-treated experimental animals. Oxaliplatin-induced cold hyperalgesia was increased by stimulation of the L-type channel, nuclear factor of activated T-cell and TRPM8, although the cold hyperalgesia was decreased by VGCC blocker treatments. In addition, TRPM8 mRNA and protein expression levels in the L4-6 DRG of oxaliplatin treated rats were increased following oxaliplatin treatment (Kawashiri et al., 2012).

### TRPV1

TRPV1 is a member of vanilloid subfamily of the TRP superfamily. The channel was firstly expressed in rats through activation of high temperature and pungent hot chili pepper component (capsaicin) in mice DRG (Caterina et al., 1997). In addition to capsaicin and high temperature (>43°C), the channel can be activated by different stimuli including low pH (<5.9), oxidative stress leading to the perception of pain, and oxidative injury (Tominaga et al., 1998; Yoshida et al., 2006; Nazıroğlu, 2015). Apart from mice, the channel was also expressed in DRG of different mammalian animals and human (Hayes et al., 2000), and also has six transmembrane domains. Cysteine groups as a source of thiol redox system act as the main source of different antioxidants such as GSH, glutathione peroxidase and alpha lipoic acid (Sen and Packer, 2000). Hence, the cysteine groups represent the main target of ROS and reactive nitrogen species (RNS) (Nazıroğlu, 2007). In addition to TRPA1 (Takahashi et al., 2011), it was reported that oxidative alterations of multiple Cys residues in different cells are involved in this mode of TRPV1 activation through modifying the extracellular (Yoshida et al., 2006) or intracellular Cys residues (Chuang and Lin, 2009) and disulfide bond formation (Wang and Chuang, 2011). In addition, results of a recent study indicated heterogeneous subunit composition of TRPV1 through heterogeneous modification of Cys-258 residues in the human TRPV1 tetrameric complex in disulfide bond of the channels (Ogawa et al., 2016). Therefore, the TRPV1 is activated in DRG (Nazıroğlu et al., 2013), hippocampus (Övey and Nazıroğlu, 2015) of rats by depletion of intracellular GSH, although the channel was inhibited in cells following treatment with thiol redox cycle members such as GSH, selenium and N acetyl cysteine (Nazıroğlu et al., 2013, 2014; Kahya et al., 2017).

ROS are produced in physiological levels as part of normal mitochondrial function and phagocytic activity. During the removal of bacteria and viruses, ROS are produced by anti-inflammatory cells such as macrophages microphages and microglia. Therefore, there is direct relationship between increased levels of ROS and inflammatory hyperalgesia (Oehler et al., 2017). Interaction between TRPV1 and long sustained thermal hypersensitivity (but not mechanical hypersensitivity) in oxidative stress-induced inflammatory hyperalgesia of the mouse hind paw has been previously reported (Keeble et al., 2009). Therefore, there is a direct role of ROS through activation of TRPV1 on hyperalgesia in the DRG neuron (Ibi et al., 2008).

Cisplatin-induced TRPV1 channel expressions were investigated in DRG neuron by Hori et al. (2010) and they observed no change on the frequency of TRPV1-positive cells in DRG neurons with different diameter by cisplatin treatment. The roles of mechanical hyperalgesia in TRPV1 knockout mouse and pronociceptive role of TRPV1 in mild burn (51°C for 15 s) injury was also reported in another study (Bölcskei et al., 2005), but they observed no pronociceptive role of TRPV1 in cisplatin-induced toxic neuropathy.

Reports on cisplatin-induced thermal sensitivity in rodents are conflicting. For example, cutaneous mechanical allodynia and hyperalgesia but not noxious thermal sensitivity was reported by cisplatin treatment (Hori et al., 2010). Some studies reported induction of mechanical and cold stimuli hyperalgesia and allodynia associated with minor motor disorders (Authier et al., 2003), whereas other studies (De Koning et al., 1987; Tredici et al., 1999) reported no effects in the responses to thermal stimulation after cisplatin treatment. In a recent study (Zhao et al., 2012), TRPV1-mediated nociceptive behaviors are not affected by cisplatin, paclitaxel and oxaliplatin. In addition, the numbers of capsaicin-sensitive DRG neurons were not changed by oxaliplatin treatment and the authors concluded that there is no role of TRPV1 on oxaliplatin-induced acute peripheral neuropathy in the DRG neurons. Consistent with the report, induction of thermal hyperalgesia through increased TRPV1 expression in the DRG after paclitaxel treatment was observed, although the hyperalgesia was decreased by TRPV1 treatment (Hara et al., 2013). In addition, the TRPV1 activator role of paclitaxel via stimulation of TLR4 signaling was reported in DRG neurons of human and paclitaxel-treated rats (Li et al., 2015).

In a study, diameters of TRPV1 remained unchanged in mice DRG neurons following cisplatin treatment, although the occurrence of TRPV1 in the neurons was increased by cisplatin treatment (Khasabova et al., 2012). In contrary to the report, no protective effect of TRPV1 (AMG9810) or TRPA1 (HC030031) antagonists on cisplatin-evoked mechanical and cold allodynia in rats was reported in another study (Guindon et al., 2013). Induction of mechanical hyperalgesia, and cold allodynia (via 10°C water) in rat models of cisplatin-induced peripheral neuropathy were reported (Authier et al., 2003; Nassini et al., 2011). Similar result was observed by Ta et al. (2009) and increased thermal hyperalgesia to cold was reported in cisplatin-treated mice. However, some authors attributed the direct effect of cisplatin to TRPA1 instead of TRPV1 in the neuron, because TRPA1 receptors are required for the development of cisplatin-evoked mechanical allodynia in mice (Nassini et al., 2011; Khasabova et al., 2012).

Increased intracellular Ca^2+^ concentrations induced release of excessive substance P from the central and peripheral nerve terminals of DRG neurons in response to noxious stimuli (Sacerdote and Levrini, 2012). The role of VGCC blockers and TRPV1 channel was also investigated on paclitaxel- and vinorelbine (a chemotherapeutic drug)-induced substance P release in DRG neuron of rats and no role of TRPV1 on the substance P release was observed in the DRG (Miyano et al., 2009).

The involvement of oxaliplatin on the release of calcitonin gene-related peptide from rat sensory neurons in culture was recently reported (Pittman et al., 2014). In addition, they reported that TRPA1 and TRPV1 channel activation-induced transmitter release were increased or decreased according to the concentration and exposure time of the drug and in peptidergic DRG neurons with small diameter by paclitaxel treatment.

### TRPV2

Another member of TRP superfamily is the TRPV2 and the channel is also a member of thermosensitive TRP channels and it is activated by a very high-threshold heat temperature (>52°C; Ahluwalia et al., 2002). There are limited data and reports on the physiological role of the TRPV2 channel in the literature. Cisplatin-induced TRPV2 channel expressions were investigated in DRG neuron (Hori et al., 2010) and increased of TRPV2 protein expression in the small-cell of L5 positive DRG neurons but not in L5 DRG cells innervating gastrocnemius muscle was reported following cisplatin administration (Hori et al., 2010). Increase of highly noxious temperatures (>56°C)-induced TRPV2 protein expression levels in peripheral thermal of neuron via the transduction of pain hypersensitivity (Shimosato et al., 2005). Because selective TRPV2 antagonists are not commercially available, further mechanistic studies including TRPV2 knockout mouse might be needed to determine the exact involvement of TRPV2 in cisplatin-induced neuropathy.

### TRPV4

As a member of TRP superfamily, TRPV4 was firstly described with mammalian osmotransducer property (Liedtke et al., 2000). Several activators of TRPV4 such as low pH, citrate, phorbol esters, arachidonic acid, oxidative stress, and exogenous chemicals (bisandrographolide A) have been described (Güler et al., 2002; Alessandri-Haber et al., 2004; Materazzi et al., 2012). Additionally, TRPV4 is activated by heat (24°C≥) (Güler et al., 2002) and the channel is also a member of thermo-TRP group. Enhanced nociception in neuropathic pain was reported by heat activation of TRPV4. Therefore, TRPV4 is essential for inflammatory thermal hyperalgesia (Davis et al., 2000), but not for normal heat sensation (Caterina et al., 2000).

Induction of hyperalgesia through activation of α2β1 integrin and Src tyrosine kinase pathways in rat DRG neuron was reported in the TRPV4 knockout mice by paclitaxel treatment (Alessandri-Haber et al., 2008). However, similar results were not shown in hind paw and DRG of rats by the same study, and TRPV4 did not act an essential role in paclitaxel-induced nociceptive behavioral responses to mechanical and hypotonic stimulation in the hind paw (Alessandri-Haber et al., 2004). It was reported in DRG neurons of wild type and TRPV4 knockout mice that TRPA1 and TRPV4 are activated by paclitaxel-induced mechanical allodynia and excessive ROS production but not cold exposure, although the allodynia and oxidative stress was partially decreased by treatment with a TRPV4 (HC-067047) antagonist (Materazzi et al., 2012). RN1734 is also a TRPV4 antagonist (Vincent et al., 2009) and inhibition of TRPV4 did not alter nociceptive baseline in control mice, and mechanical allodynia and heat are partially reserved by RN1734.

## Conclusion and future subjects

Accumulating evidence suggests that neuropathic pain and painful neurotoxicity in the rodents are increased by selected chemotherapeutic agent through increased sensitization of TRPA1, TRPM8, and TRPV1. In addition, antagonists of TRPA1 and TRPM8 were able to attenuate cisplatin, oxaliplatin, and paclitaxel-induced mitochondrial oxidative stress, inflammation, cold allodynia, and hyperalgesia, although TRPV1 was responsible for cisplatin-induced heat hyperalgesia and mechanical allodynia in sensory neurons. TRPA1, TRPM8, and TRPV2 protein expression levels were mostly increased in the DRG and trigeminal ganglia neurons by chemotherapeutic agents. There is a debate on direct or oxaliplatin-induced oxidative cold stress dependent TRPA1 and TRPV4 activation in the DRG. Involvement of molecular pathways such as cysteine group, GSH, anandamide, cAMP, lipopolysaccharide, proteinase-activated receptor 2, and mitogen-activated protein kinase were also indicated in oxaliplatin and paclitaxel-induced cold allodynia. Therefore, there is growing evidence for the potential role of TRP channel inhibitors as modulators of chemotherapy-induced neuropathic pain in the clinic.

A new member of the TRP superfamily is TRPM2. The enzyme ADPR pyrophosphatase in the C-terminal domain of TRPM2 is sensitive to ROS and RNS (Wehage et al., 2002; Nazıroğlu, 2007; Nazıroğlu and Lückhoff, 2008). It is well-known that excessive ROS production contributes to sensitization in persistent pain of DRG neuron (Kallenborn-Gerhardt et al., 2012). In addition, results of recent studies have suggested the involvement of warm temperature on the activation of TRPM2 channels in the rat DRG neurons (Tan and McNaughton, 2016). To our knowledge, there is no study of the interaction between TRPM2 channel and chemotherapeutic agents in DRG neurons. Future studies should investigate the interactions between TRPM2 and other oxidative stress-dependent TRP channels such as TRPM7 and TRPC5 in the DRG neuron following exposure to chemotherapeutic agents. There is no report on interactions between remaining thermo-TRP channels such as TRPV3 and TRPM3 and chemotherapeutic agents in the peripheral neurons. The interaction should be also clarified in primary neurons.

## Author contributions

MN formulated the present hypothesis and he was responsible for writing the report. NB made critical revision for the manuscript. The original figures were produced by MN.

### Conflict of interest statement

The authors declare that the research was conducted in the absence of any commercial or financial relationships that could be construed as a potential conflict of interest.

## Abbreviations

[Ca^2+^]_i_: intracellular free calcium ion
CAPS: capsaicin
CHO: Chinese hamster ovary
CPZ: capsazepine
DRG: dorsal root ganglion
fMLP: N-formylmethionine peptides such as formylmethionyl- leucyl phenylalanine
LPS: lipopolysaccharide
MAPK: mitogen-activated protein kinase
PAR2: proteinase-activated receptor 2
PARP-1: Poly-ADPR polymerase 1
ROS: reactive oxygen species
TRP: transient receptor potential
TRPA1: transient receptor potential ankyrin 1
TRPM8: transient receptor potential melastatin 8
TRPV1: transient receptor potential vanilloid 1
TRPV4: transient receptor potential vanilloid 4.
